# Preventing suicides on the railways: learning from lived and living experiences

**DOI:** 10.1186/s12889-025-22744-x

**Published:** 2025-05-02

**Authors:** Jay-Marie Mackenzie, Ian Marsh, Bob Fields, Ian Kruger, Dafni Katsampa, Ioana Crivatu, Lisa Marzano

**Affiliations:** 1 University of Westminster, London, UK; 2 Canterbury Christ Church University, Canterbury, UK; 3 Middlesex University, London, UK; 4 University of Hertfordshire, Hatfield, UK; 5 University of Suffolk, Ipswich, UK

**Keywords:** Suicide, Railway, Prevention, Lived Experience, Living Experience

## Abstract

**Background:**

Despite increasing recognition of the crucial role of lived/ing experience in shaping suicide prevention policy and practice, the perspectives of people who have considered or attempted suicide by train are seldom captured in analyses of what could reduce suicides on the railways. The aim of this study was to explore lived/ing experience perceptions of what types of approaches are effective or ineffective in this context, and why.

**Methods:**

We carried out 1) in-depth qualitative interviews with 34 individuals who had attempted or contemplated suicide on the railways; 2) an online survey investigating lived/ing experiences of suicidality at rail locations (*N* = 269); 3) an online ethnography of content relating to train/rail suicide from different online spaces including ‘pro-choice’ forums and reddit (254 posts and 1228 associated comments).

**Results:**

Several measures to prevent suicide on the railways were identified—and critiqued—in lived/ing experience accounts. These included strategies to challenge dominant cultural narratives around railway suicide (e.g. by shifting the focus from the lethality of this method to its impact on train drivers and others); environmental measures to restrict access to means and/or create a safer and more positive atmosphere; and increasing opportunities for help-seeking and ‘helpful’ third-party interventions. However, considering what works for whom, and when, emerged as crucial. The challenges of preventing rail suicides against a backdrop of severely stretched mental health services were also repeatedly highlighted.

**Conclusions:**

The perspectives of people with lived/ing experiences, whilst far from homogenous, provide crucial insights into the potential value and unintended consequences of different measures to prevent suicides on the railways. Our findings reiterate the need for comprehensive suicide prevention strategies, targeting different stages of the suicidal process.

**Supplementary Information:**

The online version contains supplementary material available at 10.1186/s12889-025-22744-x.

## Background

A vital element of any suicide prevention work is to include expert voices such as those with lived/living experiences [1–3], yet this is often missing in the literature [4, 5]. Experts by lived/ing experience can include suicide attempt survivors, those who live or have lived with suicidality, and/or have lost a loved one to suicide. Their voices, whilst not homogenous [6], allow us to understand a range of issues linked to the effectiveness of preventative measures, including questions around feasibility, acceptability and potential for harm or other unintended consequences [7]. This is especially important in complex environments, such as the railways, in which there has been limited evaluation of preventative approaches [8].

In many countries, suicides on railways and subway rail systems constitute a substantial proportion of overall suicide numbers (e.g., around 1 in 20 suicides in England [9]). Suicide prevention on the railways involves restricting access to the site and the means of suicide, mostly through the use of platform screen doors and open track barriers; increasing opportunities and capacity for human intervention (for example, in the UK, many railway employees are trained to look out for and offer support to people who may be suicidal); increasing opportunities for help-seeking by the suicidal individual through the use of messaging on the railways (in the UK, Network Rail work in partnership with Samaritans and other charities within the wider community to de-stigmatise suicide and promote help-seeking behaviour); and reducing the ‘cognitive availability’ of railway suicide by, for example, ensuring responsible media reporting. However, strategies to reduce access to means, whilst seemingly (also) effective in this context [8, 10], may not always be feasible or cost-effective in relation to large rail networks [11]. Few other measures to reduce suicides by train have been formally evaluated, even in countries such as the UK which have targeted multi-level strategies for suicide prevention on the railways [12]. The most recent published review of evidence-based rail interventions identified only nine studies [8], of which only four focusing on measures other than platform screen doors or ‘suicide pits’: three studies suggesting that blue lights “may be effective suicide prevention strategies” (but with perhaps limited impact [13]) and a 1998 paper demonstrating the effectiveness of changes in news reporting of subway suicides in Vienna [14]. More recently, research in Denmark has shown that a combination of messaging, physical barriers and motion-sensitive lights may be beneficial at stations [15], but further evidence is needed to establish the impact of these measures or their effectiveness in other railway environments.

Previous work with people who have considered or attempted suicide at rail locations has provided important insights into why and how people might choose these locations [5, 16, 17]. However, much of the work focussed on expert knowledge of what is effective (or ineffective) to reduce suicides on the railways has engaged the views of stakeholders implementing suicide prevention at these sites [18, 19]. Currently we know very little about what people with lived/ing experiences of suicide think about suicide prevention in these environments, including their perceptions about which types of prevention are likely to be effective or ineffective, when, why and for whom. There is also little evidence of how effective these forms of prevention may be for people at different stages of the suicidal process [20], or with different views and experiences of suicide prevention and interventions. For example, some people may be open to interventions whilst others may not [6].

Therefore, the current research aimed to capture the perspectives of a range of people with lived/ing experience on suicide prevention within rail contexts, including views on what types of approaches could be effective or ineffective, and why.

## Methods

### Design

We carried out three studies focused on lived/ing experiences of rail-related suicidal thoughts and behaviours (including at station and non-station locations, on both mainline/overground and metro/underground networks): 1) qualitative interviews with people who had attempted or contemplated suicide on the railways; 2) an online survey investigating lived/ing experiences of suicidality at rail locations; 3) an online ethnography of content relating to train/rail suicide. Using these different approaches allowed us to get a range of lived/living experience perspectives and to explore nuances within these accounts, as well as foregrounding voices not usually accessed through traditional research methods [6].

#### In-depth interviews

Through a national online survey (see [16] for further details), we identified 34 UK-based individuals who had considered or attempted suicide on the railways and expressed an interest in participating in a follow-up interview. This included people who had survived an attempt on the railways (‘group A’; 7 males and 3 females); participants who had survived an attempt by another method, having considered but then rejected a rail suicide (‘group B’, 3 males and 11 females); or experienced thoughts of rail suicide but not made a suicide attempt (‘group C’, 2 males and 8 females). A semi-structured interview schedule was used to elicit experiences of suicidality on the railways, including perceived triggers and motivations, as well as barriers against railway suicide which had or could influence decision-making, and thoughts on preventative measures.

All participants were aged 18 or over and gave written informed consent before taking part in telephone (*n* = 17), email (*n* = 11) or face-to-face (*n* = 6) interviews (see [5] for full details).

#### Online survey

As part of a wider study of suicidality and life-saving interventions, we gathered the views of 269 individuals with lived/ing experience of suicidal ideation (*n* = 240) or attempts (*n* = 29) involving the railways, on “what the rail industry can do to prevent suicide attempts on the railways”, “what could make things worse” and, where applicable, what had prevented them from attempting suicide by this method (with no prompting, structure or limit to the answers that could be provided; see supplementary material).

All participants were over the age of 16 (median age = 32; range = 16–74), most described themselves as White British (149/187, 79.7%), and all but 14 (5.2%) lived in the UK at the time of completing the survey. Just over half those who provided information about gender described themselves as male (99/186, 53.2%; 77 (41.4%) identified as female, 2 (1.1%) as transgender and 8 (4.3%) as non-binary).

The study was advertised online (particularly, but not exclusively, within special interest groups and networks with a focus on suicide prevention, mental health and UK railways) and via posters and leaflets on university campuses and at busy railway locations, between April and October 2019. Links to further information about the study, and to support services for those experiencing suicidal thoughts, were available both at the beginning and the end of the survey (see [21] for further details).

#### Online ethnography

Data were collected from ‘pro-choice’ suicide forums and online spaces where people discuss suicide and posts from reddit surrounding the topic of suicide between 14th December 2018 and 14th December 2019 (total 254 posts and 1228 associated comments) using search terms relating to suicide and railways (see [6]). These online spaces were chosen as a source of data as they are widely used (particularly by UK-based users), are available on the ‘clearnet’ (as opposed to ‘darknet’) and are moderated. The online spaces varied on the level of discussion about suicide methods allowed on the site, with some sites prohibiting discussion of methods and others allowing such conversations. These different sites thus provide discursive material on different methods of train suicide; generated by people who might not normally get involved in suicide prevention research but whose insights can be very valuable. Whilst the conversations on all online spaces covered a wide array of topics, from survival stories to discussing different suicide methods, the search strategy focused on highlighting those where the primary conversation was about railways.

### Analysis

Each study was first analysed independently:

1. Interviews were audio-recorded, transcribed verbatim and analysed for semantic and latent themes using an inductive approach based on reflexive thematic analysis [22]. Data coding was iterative, with coding and final themes checked for consistency by two researchers using NVivo V.10.
2. Open-ended survey data were analysed inductively for content [23]. Approximately 10% of responses were coded by three researchers, for inter-rater reliability and coding scheme refinement. Frequencies and percentages, as appropriate, were then used to summarise participants’ responses, alongside anonymised survey quotes.
3. Online posts relating to rail suicide were analysed thematically, to identify patterns of meaning within the texts and fieldnotes. An iterative process of open, axial, and selective coding was used. Each post was initially coded to capture its content and focus (with more than one code being assigned to many posts); then reviewed in the second stage of analysis, when broader themes were assigned to posts that consolidated the open codes.
  Finally, selective codes were identified that represented the central or main themes. In addition, emerging patterns of interaction in the discussions were noted; that is, the ways in which particular ideas and arguments, points of view, and expressions of emotion recurred within and across threads.

The final stage of the analysis involved the wider research team integrating interview findings with those from the survey and online ethnography. This involved an iterative, ‘following a thread’ approach [24] with key themes from the interviews being followed across the online survey and then the online ethnography. Any complementary or discrepant findings were noted and, by team consensus, were included in the final write-up where deemed to add to the understanding and prevention of railway suicide.

#### Ethics and lived/ing experience involvement

The research was approved by the Psychology Research Ethics Committee at Middlesex University (ref: ST019-2015 for study 1; 7045 for studies 2 and 3). All research materials and procedures adhered to the World Medical Association’s Declaration of Helsinki and were developed in consultation with Samaritans, who commissioned this work on behalf of the rail industry, and with an expert advisory group, which included individuals with lived/ing experience of suicidality and suicide bereavement, and which also assisted the researchers in refining key findings and considering their implications. Identifiable information was removed from all datasets. To protect the privacy of individuals whose online posts were analysed, we have not named the sites used or included direct quotes, but paraphrased users’ comments to illustrate the main themes of discussions.^1^

### Findings

Several measures to prevent suicide on the railways were identified—and critiqued—in lived/ing experience accounts, both for people who had already taken steps towards this method (e.g., at a station) and in the months/weeks/days leading up to a suicidal act (see Table 1). Our findings suggest that considering what works for whom, and when, is crucial.

**Table 1:** Overall themes

Themes	Subthemes
Shifting Associations about Rail Suicide	*Careful Communications* *Deterrence: Survivor Stories and Impacting Others*
Environment-Based Prevention	*Creating a Positive Atmosphere* *Restricting and Reducing Access*
Increasing Opportunities for Help-Seeking	*Visible and Accessible Support Resources* *Time-Critical Prevention*
Third-Party Interventions	*Increasing the Likelihood of Detection* *Human Intervention and Presence* *The Importance of Helpful Interventions*
General Mental Health Support – the Bigger Picture	

### Shifting associations about rail suicide: careful communications

Both interview and survey participants reported that messages about suicide and (high) lethality were frequently conveyed, often unintentionally, in and around railway contexts. Over time these messages were said to strengthen associations between the railways and suicide, and therefore the cognitive availability of trains as effective means of suicide (i.e., *“it promotes copycats and gives a false impression of the effectiveness of the method” (Survey Respondent (SR) 97).* News and announcements of rail suicides (and related service disruptions) should therefore be done sensitively:

*“I'm not saying pretend it doesn't happen, but much, much more care needs to go into how these things are portrayed” (SR 187)*

*“It’s in our paper every week really I think, ‘There’s been a fatality at wherever.’ So obviously that’s very prominent in your mind. But I think it’s fuelling itself. I think people are seeing, ‘Oh well, yeah okay, that succeeded. I’m in a desperate state, I’ll do that too.’ (Group A Interviewee (‘A’) 4)*

*“Please stop announcing suicides so explicitly at stations. Say something like there’s a medical emergency down the line” (SR64)*

Some participants also stressed the importance of “*not making it sound like an inconvenience” (SR14)* when trains are delayed due to a suicide, and to also be cautious in communicating about wider risks at rail locations (e.g., in relation to accidents and trespass):

*“The automated announcements […] that a train would be passing through the station at speed, which is what triggered my impulse to jump. The opportunity was presented without having prior consideration for it. I should note I find it quite ironic that a safety instruction had conveyed a meaning to me that was quite the opposite to its literal intent.” (C1)*

Announcements such as these could retain messages about risk and safety, but *“highlighting the support rather than the dangerousness, I suppose, of the situation.” (B13).*

#### Deterrence: survivor stories and impacting others

An overwhelming consensus amongst participants was that railway attempts are almost always considered to be lethal, yet this is not always reality and life-changing injuries may instead occur. Linked to this theme was the suggestion to *“educate on the stats of efficacy of attempts (probably not as high as we think*); *show personal stories” (SR 170):*

*“People do survive… If people could hear from survivors… just to hear stories of what people have been through and how they are now… I think it would be really helpful”. (B4)*

*“You wouldn’t want to be left mangled and still alive because that would be even worse. I don’t think that’s made clear at all. In fact I don’t think I’ve ever heard that.” (A4)*

Indeed, when asked what had prevented them from acting on thoughts of rail suicides, 32 (17.9%) survey respondents mentioned fear, including of the possibility of survival with injury (see Table 2), hence *“making it widely known that it is likely to be extremely painful and not effective may deter attempts.” (SR22).*

**Table 2:** What prevented you from attempting suicide on the railways?

	n/179*	**%**
**1. Psychological Factors**		
Consideration of impact on others (rail staff, commuters and other witnesses)	76	42.46%
Consideration of impact on loved ones	29	16.20%
Overcoming suicidal crisis/desire to live	30	16.76%
“Chickening out”/fear	19	10.61%
Fear of survival/injury	13	7.26%
Religiosity	1	0.56%
Suicide by train not preferred method	9	5.03%
**2. Environmental Factors**		
Third-party intervention, involving:	17	9.50%
• Rail staff/police	4	2.23%
• Commuter	6	3.35%
• Loved ones (including 4 serendipitous calls/encounters)	8	4.47%
Not sufficiently private/likelihood of intervention	10	5.59%
Train arrived/did not arrive	2	1.12%
Too loud	1	0.56%
Other	2	1.12%
**3. Other**		
Receiving professional support/medication	6	3.35%
Support from loved ones	2	1.12%
Unsure	3	1.68%

Overall percentage is > 100 as some participants gave multiple responses

^*^Not all participants who reported having considered suicide by train or whilst at a rail location (*N* = 240) provided an answer to this question

An even more common deterrent was the impact of rail suicides on other people, especially train drivers and other witnesses (as reported by almost half the survey participants who commented on their reasons for not acting on thoughts of train suicide (76/179, 42.5%)). Therefore*, “posters showing the devastation caused, the trauma to the driver involved, the human impact on the survivors—might be more effective. I’m glad I didn’t ruin a driver’s life” (SR39).*

These positions were also echoed in online forums. Suicides by train were often described online as “*selfish”*, and this raises questions as to whether campaigns which attempt to reduce stigma around suicide, or which explicitly declare suicide not to be a selfish act, may have unintended consequences. Involving others in one’s suicide was often looked upon very negatively in posts, with the potentially traumatic effect on the driver particularly prominent as an argument against the method. Similarly, the other main deterrent stated was in relation to experiencing pain, the method not always being lethal, and surviving with injuries, which raises questions as to what the effects would be of having these elements more frequently talked about. These arguments and responses were often relayed time and time again in threads (albeit in different ways).

### Environment-based prevention: station atmosphere and means restrictions

Improvements to the built environment were frequently suggested by interview and survey participants, both *“the subtle things to make some stations a bit more calming maybe” (e.g. plants, music and comfortable seating areas away from the tracks to "make the experience less dark, detached and dingey” (SR43))* and, crucially, to restrict access to (fast) trains *(“currently it is very easy to access the tracks” (SR48)).* The latter was the single most common preventative measure recommended by survey respondents (Table 3) and included physical changes to prohibit a person from accessing the location in the first place (e.g. *“anti-climb paint” (B3); “higher fences or something like that” (A5))* and structural solutions to prevent access to the tracks, especially for high-speed lines.

*“High-speed lines if passing through stations ought to be inaccessible by passengers if at all possible.” (C1)*

*“I would put the access to the fast lines at the furthest point away from the entrance so you have to go past a lot more people to get there… because I was conscious of not being seen by people, so that might have prevented it” (B1)*

**Table 3:** What can the rail industry do to prevent suicide attempts on the railways?

	n/159*	**%**
**1. Means Restriction**		
Barriers/doors/fencing, of which:	58	36.48%
• At stations/on platforms	31	19.50%
• Lineside	15	9.43%
• On rail bridges	6	3.77%
• At level-crossings	3	1.89%
• Unspecified (e.g. ‘barriers’)	11	6.92%
- BUT difficult/unfeasible/prohibitive	9	5.66%
Other environmental interventions to reduce access to means:		
• Slowing down trains as they approach stations	6	3.77%
• Modifications to trains	2	1.26%
**2. Increase Likelihood of Intervention**		
Increase/improve staffing	33	20.75%
Staff training	17	10.69%
Increase public awareness/education	17	10.69%
Cameras, security, improved monitoring	8	5.03%
**3. Communications and Signage**		
Dissuasive messaging highlighting:		
• Impact on others (especially driver, family, bystanders)	13	8.18%
• Possibility of survival with injury/challenge lethality	8	5.03%
• Impact on body	2	1.26%
Reduce cognitive availability (e.g. via announcements)	4	2.52%
Signage, of which:	30	18.87%
• Crisis signage with helpline contacts (e.g. Samaritans)	25	15.72%
• Messages of hope/support	4	2.52%
• Highlighting impact on others	2	1.26%
• Encouraging intervention/reaching out	1	0.63%
• Humorous ads	1	0.63%
**4. Station Atmosphere and Design**		
Stations generally nicer/friendlier	6	3.77%
Safe/quiet spaces/seating away from tracks	6	3.77%
Blue/mood stabilising lights	4	2.52%
More/better alert/help points and mechanisms to alert staff	4	2.52%
Music	3	1.89%
Plants/flowers	3	1.89%
Sponsored help telephones away from platforms	1	0.63%
Wider platforms	1	0.63%
Writing on platforms (e.g. messages of hope)	1	0.63%
Distracting noises	1	0.63%
Easy way to get off the tracks for people who have changed their mind	1	0.63%
**5. Other**		
Nothing/very little can be done	27	16.98%
Unsure	10	6.29%
The rail industry is doing a lot/the right things	10	6.29%
Not the rail industry’s fault/responsibility	8	5.03%
Nothing *should* be done to prevent (my) suicide	4	2.52%
Importance of multi-agency approach	4	2.52%
Need/suggestions for targeted strategies (e.g. focus on quiet locations)	17	10.69%
Other	4	2.52%
**What could make things worse?**	**n/119**^**a**^	**%**
Unhelpful interventions/interactions (with staff, police or others)	23	19.33%
Unstaffed/understaffed locations	11	9.24%
Announcements/reporting after a suicide, including:	11	9.24%
• ‘Highlighting locations’ and method	5	4.20%
• ‘Trying to cover suicides/fatalities up’	5	4.20%
• ‘Making it sound like an inconvenience’	1	0.84%
• Using the word ‘committed’	1	0.84%
Announcements/campaigns/talk about suicide prevention	4	3.36%
Signage	6	5.04%
Other environmental factors, including:	19	15.95%
• Atmosphere (e.g. noise, tension, silence)	5	4.20%
• Weather conditions	2	1.68%
• Lack of measures:		
◦ No signage	3	2.52%
◦ No fences	2	1.68%
◦ No staff training	1	0.84%
◦ No monitoring	1	0.84%
• Digital screens (e.g. “large screens with depressing news”)	2	1.68%
• Non-stopping trains	1	0.84%
• Other	4	3.36%
Wider societal/financial/funding issues	6	5.04%
Not doing anything/more	4	3.36%
Nothing/not much could make things worse	12	10.08%
Unsure	22	18.49%
Other	4	3.36%

Overall percentage is > 100 as some participants gave multiple responses

^*^Not all participants who reported having considered or attempted suicide by train (*N* = 231) provided an answer to these questions

Interview and survey participants also stressed that, even when not very high, physical barriers can serve as a psychological barrier, by increasing the perception that access would be difficult (“*almost like a visual deterrent to put people off” (SR139))* – if well-maintained:

*“If the barriers aren’t broken or damaged or anything, you might think that people are around there more often. [Otherwise, I’d think] “Oh look, that’s really easy to break. If I wanted to get in front I could.” (B13)*

Yet restricting access to these environments was not considered a clear-cut solution. Several participants commented that although environmental changes may delay suicide, the person may move onto another location or use another means:

“*Whether you’re just going to drive around and find one that isn’t well lit I don’t know; but obviously it’s certainly going to delay you to a degree. (B10).*

Participants also expressed some practical and feasibility concerns:

*“We can’t live in a country where let’s just brick everything up and block everything off because they’ll just find another way.” (B12)*

*“Enclosing lines and shutter doors at all stations – this would be prohibitively expensive” (SR4)*

Interestingly in many forum discussions the railways were seen as a last option when other methods had been exhausted. User comments echoed interview and survey participants’ views on method accessibility and availability, and there was a significant amount of discussion relating to *“*c*ommuting and proximity to the tracks”.* Proximity was central to several posts when discussing this topic; people knew how far away the nearest tracks were both in time and distance. This indicates the degree of knowledge and planning that people have about the location of quieter points on the tracks. It also demonstrates the association that some may have on their daily commutes of train stations being potential places to take their own life [9]

### Increasing help-seeking opportunities: visible support

In participants’ accounts, information focused on ‘where to get help’ could be effective for individuals in the early stages of the suicidal process, i.e. those who had not progressed towards planning their suicide, but had thoughts about rail suicide:

*“I remember sort of sighing to myself because I saw the Samaritans’ posters after I’d tried everything, but if people do see them beforehand then it’s helpful just knowing that there’s that option available for someone to reach out to.” (A5)*

*“Sometimes all a person needs is to see the right information at the right time, something that makes them pause and reconsider.” (SR2)*

An important aspect of help-seeking in railway environments involved the opportunity to do so discreetly. Technology was regarded a particularly useful tool for this purpose. For example, some interview participants suggested the use of QR codes or email/text services to prevent being heard by others or seen as visibly distressed on the phone.

*“I think we need online support services advertised in this location… It is very hard to talk aloud sometimes especially in busy locations.” (C1)*

The importance of visible and accessible help-seeking opportunities was also repeatedly stressed, e.g. details on support “*on the app to buy train tickets*” or on “*painted quotes on the floor*” *(SR126)*:

“*They should have great big billboards within the entrance of the train station with big bright bald letters with helpline numbers to call like Samaritans etc and letting people know that there are people that care and there are people that will always listen to them.” (SR132)*

Survey respondents also suggested that posters which “*encourage people to talk to each other"* could help:

*“More visible signs up of numbers to call if you feel you want to end your life. Signs to educate people what to do if they suspect someone might be actively suicidal.” (SR45)*

#### Time-critical prevention

However, visible support opportunities which rely on the person in distress accessing help were not considered effective by or for everyone: *“a poster there is just a little bit too little too late kind of thing*” *(A7)*.

*“I always say I could have walked down a corridor of wall-papered Samaritans posters and it wouldn’t have done a single thing in my mind because I was, my mind was in such turmoil at that point, when I thought about suicide it, it…in my frame of mind I don’t think that would have actually worked.” (B1)*

A few survey respondents also felt that ‘crisis signage’ can be unhelpful (Table 3): “*if anything, it gives people the idea.” (SR28).*

*“Well-meaning signage that actually just highlights effective suicide spots” (SR56)*

*“Quotes that try to help sometimes make me feel worse” (SR58)*

In the online forum, there was often scepticism expressed towards initiatives to promote help-seeking, particularly if the person was in the midst of a suicidal crisis. For example, in one discussion the printing of a helpline number on the reverse of a train ticket was criticised as ineffective, though other discussions pointed to how helpful helplines could be.

### Third-party interventions

Third-party interventions were commented upon in different ways – as a reason for not going forward with a suicide plan; as something to avoid; as something that could be helpful if done sensitively; as something that could make things worse; and something that necessitates training.

A common theme across all studies was the important role of third-party interventions for those who are actively contemplating suicide and have entered an accessible railway location (e.g. 15% of survey participants failed to act on thoughts of rail suicide because someone intervened or could have done; see Table 2) – and people’s efforts to avoid these as part of their suicide plans. Indeed, online discussions focussed heavily on how to avoid detection in these environments, for example by carefully scouting locations and taking advice from others on forums about time of day, what to wear, how to act, and so on.

Decreasing the capacity for suicidal individuals to move through the railway environment undetected was therefore felt to potentially increase the likelihood of an intervention taking place, such as staff making use of existing closed-circuit television (CCTV) to spot individuals in distres﻿s.

*“Like they have cameras everywhere down there. So if they can spot behaviour like that and think maybe someone’s in trouble. I think like they could probably keep an eye out for warning signs*” (A8)

*“More CCTV coverage at isolated foot crossings to spot someone standing on a crossing before a train is due.” (SR90)*

*“Add more visual CCTV cameras as these have been a deterrent for me.” (SR159)*

Several interview participants underlined the role of lighting in this context:

*“If things are well lit you’re not going to try and scale up. If you’re doing it like me, like you didn’t want to be seen, that’s going to put you off there. (B10)*

Above all, the presence of staff was considered crucial:

*“Maybe have more security hanging around… It's really down to people noticing if something is wrong.” (B7*).

*“Increase staffing - the visibility of someone else definitely has a discouraging impact and/or increases the chance of intervention from zero. There are many small unstaffed stations which see many fast trains go through unprotected platforms. Staffing these from first train to last would have a big impact, not to mention general safety/accessibility of rail travel to all.” (SR60)*

Several survey and interview participants also remarked on the value of other commuters being present:

“*More visibility that you’re not on your own, because that’s how you feel.” (SR44)*

*“Even if it’s someone who smiles at you, kind of thing, it might just be enough to break your thoughts away or someone that might just even come and sit next to you and just be approachable. It might be enough to break that thought pattern.” (B13)*

However, not all third party-interventions were described as helpful or effective, across all three studies. For example, survey participants pointed to *“rude”* and *“inept”* interactions and interventions as the single factor which *“could make things worse”* (Table 3; e.g. when *“people film instead of getting help or helping”* or “*Shouting. Grabbing. Telling us it will get better. Making a big scene” (SR67)*):

*"Even well-meaning individuals can quickly worsen a situation with their ignorant or uneducated words or ideas.” (SR65)*

*"They grabbed hold of me and put me in handcuffs, put me in the police van and arrested me and I was put in police cells oh for like 18 hours or something and then taken to court and I was prosecuted for it...it was pretty horrendous." (A6) *

Similarly, online posts included angry (as well as grateful) comments that an intervener had *“taken away the opportunity”*, whilst posts on ‘pro-choice’ forums called for “*people to mind their own business*, *what right do people have to stop someone?”*, “*it should be illegal to intervene”*. The latter also included negative remarks about members of the public seeing themselves as “*heroe*s” for intervening or – quite the contrary – failing to notice or help when someone is visibly distressed on a station platform.

Linked to these remarks, was the frequent suggestion amongst interviewees and survey respondents to increase and improve suicide prevention training and general awareness – for both rail employees and the public – “*on the right signs to look out for*” and "*how to talk to potential suicides in a non-confrontational way*” (SR148), because otherwise “*It would just make things worse, because then you feel like people are against you. (B7).*

*“Encourage passengers and commuters to look out for unusual behaviour, changes in appearance/mood of regular travellers. Encourage passengers and commuters to have the courage to speak to anyone who is upset - they don't have to have a solution to the problem just disrupt thought patterns and make an unhappy person feel that someone cares. It can be hard to start a conversation in a train as even busy carriages are often quiet.” (SR167)*

Related suggestions were to *“involve people with lived mental health experience in the training” (SR19)* and “a *more accessible way of letting staff know that something's up (maybe a number to text or closer "Help" buttons on the walls?)” (SR111).*

From a lived/living experience perspective, third-party interventions represent a complex aspect of suicide prevention. If done sensitively they were sometimes described as being helpful and possibly life-saving. However, they were also described (particularly online) as something to be avoided if a person was set on taking their own life, and could be unhelpful if insensitively done.

### Wider context and ‘solutions’

It is notable that several survey participants stated that rail suicides may be difficult or impossible to prevent (27/159, 16%), and that potential ‘solutions’ to this issue are much broader than the rail industry alone could implement (8/159, 5%).

*“Preventing suicide doesn't start at where life ends. We have to deal with the causes of suicide and seek to prevent or at least minimise those”. (SR118)*

In this context, the importance of improved mental health provision and general support for people in crisis “*before they get into a suicidal situation*” was a recurrent theme:

*“The rail industry is doing as much as they can (putting higher barriers on bridges, putting Samaritans' number on train tickets and the platforms), I think that it is the mental health services' responsibility to prevent suicide in the first place.” (SR133)*

Similarly, interview participants remarked that *“the barriers and the signs are not going to stop people who actually want to definitely do it. [You have to] make people aware that there’s a way to help themselves by seeing [professional] people who know how to deal with it, by getting medication and therapy…” (A5).*

## Discussion

**Fig. 1:**
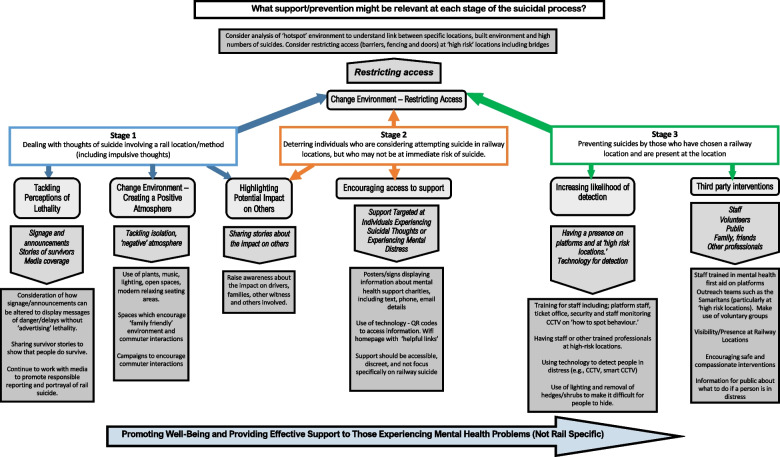
What support/prevention might be relevant at each stage of the suicidal process?

Previous research has identified which types of intervention might be useful for preventing suicides in public spaces including on the railways [8, 18, 25], however this evidence is limited and has focused on a fairly narrow range of measures [15]. For the first time, our study has considered what prevention may be helpful from the perspective of people with lived/ing experiences of suicide. Our findings provide novel insight into perceptions about what can be done to prevent such deaths for individuals at different stages of the suicidal process, including those contemplating this method, those planning/scoping a suicide at this location, and those at imminent risk of acting on thoughts of suicide by train (see Fig. 1).

Our research suggests that an important first step could be to challenge dominant cultural narratives around railway suicide, specifically that attempts by train are always lethal. This assumption appears to play an important role in decision-making around rail suicides and in increasing the cognitively availability and ‘attractiveness’ of this method [5, 16]. Challenging it in subtle ways might be helpful (e.g. sensitive signage and announcements, stories of survival and avoiding *“too much obvious ‘suicide prevention’…so there isn’t “a reminder of the reasons for doing so” (SR57)*). Shifting the association of rail suicide from lethality to its impact on others may also dissuade people from using this method, but would clearly require a careful and sensitive approach.

Another key finding is that in the earlier stages of suicidal thinking, interventions aimed at making railway environments feel friendlier and less alienating may also be helpful. Changes to the built environment to reduce access to the tracks (on rail platforms and away from stations) were described by many with lived/ing experience of rail suicidality as even more important in this context, mirroring the findings of other studies about suicide prevention in public spaces [25]. However, participants also pointed to several practical challenges to environmental adaptations in railway settings, owing to the size, function and heterogenous nature of the railway environment. Further, the insights we analysed lend support to theoretical models of suicide indicating that people may move from ideation to action when having an easily accessible method of suicide [20], and studies which highlight the role of accessibility of means in decision-making around suicide method choices [26]. However, despite other studies showing little or no evidence for displacement to other methods [8] our findings suggest that some may look for other means if they cannot access another.

One form of prevention commonly used in public spaces is the use of signs, helpline numbers, and phonelines, yet there is limited evidence as to their effectiveness [25, 27]. Our findings suggest that these may be helpful for people earlier on in the suicidal process, and should be accessible and discrete, and not specifically focused on rail suicide (to avoid reinforcing associations between the railways and suicide). However, the content and location of signage should also be carefully considered. Also, once a person had made plans and decided on a method/location, these measures were viewed as less effective. According to our findings, the most effective form of prevention at this stage is likely to involve a third-party intervention. This may include small talk or even *“just a random smile from a stranger” (SR34)* and would require the intervention to be done in a compassionate way. Training of staff was seen as particularly important here, which reflects our wider work with people who have made an intervention at a rail location [21]. Increasing the likelihood of detection and intervention in these spaces was also considered key and ways to achieve this could include technology and CCTV, increased presence of staff (and other bystanders), better lighting, and changing the environment so people can be more easily identified and call for help. Further work is needed to establish the effectiveness of these interventions for suicide prevention, and how to ensure they are implemented in safe and compassionate ways.

Finally, it is important to point out that in describing the perceived causes and possible ‘solutions’ to their suicidal thoughts and behaviours (rail-related or otherwise), many participants mentioned that *“the rail industry is doing as much as they can”* and *“the right sort of things”*, and referred to factors over which it may have limited control. Above all, frequent remarks were made about the need for better mental health services (which most interview participants and many survey respondents berated as absent and inadequate), and increased awareness and understanding of suicidality and mental health difficulties. Whilst clearly beyond what the rail industry alone may achieve, each of these suggested interventions may have railway-specific, multi-agency applications. Examples include providing mental health first aid training for staff (including transport police); posters with general information about seeking and providing support for mental health issues; encouraging personal contact at stations and ‘virtual’ networks of support [12].

### Strengths and limitations

A key strength of this research is the focus on views from people with lived/ing experiences of suicidality, something that has previously been omitted from this type of research, and compliments existing studies with stakeholders and other experts [18, 19]. The collation of insights from these three studies allowed for nuances between accounts to be explored (e.g., views on signs and posters), from a range of channels (e.g. using forums to gain opinions from people who may be less inclined to come forward for traditional research). However, there are issues regarding the generalisability of findings based on a relatively small number of cases, particularly as predominantly recruited/gathered in online spaces (albeit in different spaces and via different means). Further limitations relate to the (exclusive) focus on self-report data. With regards to online forums, there is the additional issue that participants were not responding to specific research questions. The insights gathered across these three studies may not necessarily be representative of all individuals with lived/ing experiences of suicidality on the railways, or indeed those who have died by this method. Studies of suicide deaths [9, 28, 29] and analyses of observable data (e.g. behaviours preceding a rail suicide [17, 30]) should also be considered in planning measures to reduce railway suicides.

## Conclusions

Our findings reiterate the need for comprehensive suicide prevention strategies, targeting different stages of the suicidal process: at the point when rail-related suicidal thoughts begin to form, through to the stage when suicidal ideas start becoming more consolidated into plans, and then at the point when plans or impulsive thoughts are acted upon – bearing in mind that individuals may move between different suicidal states. The perspectives of people with lived/ing experiences, whilst far from homogenous, provide crucial insights into the potential value and unintended consequences of difference strategies to prevent suicides of the railways. Further trials, designed and carried out with lived/ing experience and other experts, are needed to assess the risks, effectiveness and cost-effectiveness of individual measures and multi-level strategies to prevent suicides in railway environments.

## Supplementary Information

Supplementary Material 1.

## Data Availability

The data that support the findings of this study are available on reasonable request from the corresponding author (L.M.). The raw data are not publicly available as they include qualitative quotes that could compromise the privacy of research participants.

## Footnotes

1 There are ongoing ethical debates about the use of social media and online data in research, with questions regarding where the boundaries between ‘public’ and ‘private’ information lie [31]. In this research, online content was defined as public and therefore included in analysis if it was visible without needing to sign in. Consideration of ethics also extends to the presentation of findings. It has been suggested that this should reflect the level of confidentiality, privacy and traceability that social media users may reasonably expect [32]. Whilst participants to the discussions analysed in this study knew that posts were publicly visible, this does not mean that they necessarily consented to their words being quoted in research. In order to maintain the privacy and anonymity of site users, the sites and users are not named here. People turn to social media sites to disclose experiences in spaces where they feel a level of anonymity and safety. Therefore, it is important to maintain an expected level of privacy. Direct quotes have not been used in this report as these would be searchable, and therefore users potentially identifiable, online. We have instead paraphrased users’ comments to illustrate the main themes of discussions. Every effort has been made to ensure that the privacy of the users has not been compromised more than is necessary to illustrate the discussions which take place on these sites.
