# Supplementary material for: Preventing suicides on the railways: learning from lived and living experiences
Source: BMC Public Health. 2025 May 2;25:1625. doi: 10.1186/s12889-025-22744-x (PMC12046682; doi:10.1186/s12889-025-22744-x)
Supplement: Supplementary file 1 — Supplementary Material 1. [file 12889_2025_22744_MOESM1_ESM.docx]

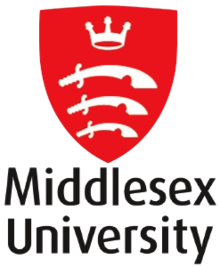


**RESEARCH INFORMATION SHEET**

**Understanding How Best To Support People In Crisis**

We are very pleased to invite you to take part in a research study that is being conducted by Middlesex University, with colleagues at the University of Westminster. This information sheet explains why the research is being conducted and what it will involve. Once you have read the information you are welcome to get in touch with the researchers if there is anything that is not clear, or if you would like more information (contact details below). You can take as much time as you like to think about taking part.

**What is the purpose of the study?**

The aim of this study is to explore and understand personal experiences of suicidal thoughts and attempts, and views about helping others who may be experiencing suicidal feelings, including family and friends, as well as strangers and passers-by. This is an important aspect of understanding how best to support those who are feeling in crisis, and we hope that it will help to reduce and prevent suicide attempts.

As part of the research we would like to hear the stories and experiences of a range of people, including those who have experienced suicidal thoughts; those who intervened in some way, or considered intervening, when somebody around them was suicidal (for example by approaching them or calling for help); and those who have never knowingly been around other people who were suicidal, or experienced suicidal thoughts themselves.

**What will happen if I take part?**

This study is being conducted online. If you do decide to take part, you will be asked some questions about your own experiences of suicidal thoughts and behaviour, as well as your experiences of, or thoughts about, intervening when somebody else is experiencing suicidal thoughts. Completing he questionnaire should take around 10-20 minutes, depending on the experiences you might be sharing.

Previous research suggests that most people have no difficulty answering these types of questions, and are pleased to be able to provide this information. However it is possible that some questions could be difficult or potentially upsetting for you, in which case we would not want you to answer them. **Before deciding to take part in this study, please consider carefully whether you are likely to find anything distressing and let us know if this is the case. Given the sensitive nature of the study, we are only inviting people over the age of 16 years to participate, and would advise against taking part in the research if you are currently experiencing strong thoughts of suicide, or have attempted suicide in the past month.**

**Do I have to take part?**

You do not have to take part if you do not want to. If you decide to take part you may withdraw from the research at any point during the study. You can also ask us to delete all of your responses within two weeks of taking part in the study (before the data analysis starts). When completing the online survey, you will be given the option to create a unique number. Please contact us at the email address provided below, quoting this number, and we will remove your data. No explanation for your request will be necessary or required.

**Will my answers be anonymous?**

Yes, all of your responses will be kept completely anonymous and confidential.

**What will happen to the results of this research study?**

We will produce a summary of the results for the charity Samaritans, who commissioned this research. The results of this study may also be published in academic journals. You will not be personally identified or identifiable in any report or publication resulting from this study.

**Who has reviewed this study?**

All research that involves human participants has to be reviewed and approved by an Ethics Committee before it can begin. The Middlesex University Psychology Department Ethics Committee has approved this project.

**Who is funding this research?**

This research was commissioned by Samaritans, on behalf of the rail industry, as part of their work to reduce and prevent suicide.

**Contacts for Further Information**

Thank you for taking the time to read this information. If you have any further questions about the study, please don’t hesitate to contact us via the email, phone or postal address shown below:

Dr Lisa Marzano

Department of Psychology

Middlesex University

Town Hall

The Burroughs, Hendon

London, NW4 4BT

Email: L.marzano@mdx.ac.uk

Tel: 0208 411 6998

You can also download a copy of this participant information sheet by pasting the following link into your browser.

**FURTHER SUPPORT**

**If you feel that you are in need of immediate support, please contact Samaritans on 116 123 or NHS Choices (**[**www.nhs.uk/111**](http://www.nhsdirect.nhs.uk)**) on 111 (both are available 24 hours a day, 365 days a year, and free). Alternatively, please go to, or call, your nearest Accident and Emergency (A&E) department and tell the staff how you are feeling.**

**For more information about helping people who may be suicidal see Samaritans' tips on**[**how to start a difficult conversation**](https://www.samaritans.org/difficultconversations) **and on interrupting someone’s suicidal thoughts using small talk (**[**https://www.samaritans.org/media-centre/our-campaigns/small-talk-saves-lives**](https://www.samaritans.org/media-centre/our-campaigns/small-talk-saves-lives)**).**

**Rethink Mental Illness also has advice on**[**how to support someone who is having suicidal thoughts**](https://www.rethink.org/carers-family-friends/what-you-need-to-know/suicidal-thoughts-how-to-support-someone) **(https://www.rethink.org/carers-family-friends/what-you-need-to-know/suicidal-thoughts-how-to-support-someone).**

**CONSENT**

Please tick the appropriate box:

☐ **YES**, I am over the age of 16 and would like to continue and take part in the study

☐ **NO**, I do not wish to continue and take part in the study

***Below are a series of questions about your experiences of suicidal thoughts and attempts. The first section asks about your own experiences of feeling suicidal, if this applies to you. This is followed by some questions about being around other people who might be suicidal, whether or not this has happened to you before (for example through family and friends, or strangers).***

PART 1: About your experiences of suicidal thoughts

**1.1 Have you ever had thoughts of ending your life?**

| No  [Go to part 2] | Yes |
| --- | --- |

**1.2 Have you ever attempted suicide?**

| Never | Once | Between 2 and 4 times | Five times or more |
| --- | --- | --- | --- |

**1.3 Have you ever been suicidal, or attempted suicide, in a public place (such as a railway station or a bridge)?**

- No [go to 1.4]
- Yes -> (please tick as many as apply)
  - - Train/tube station or tracks [go to 1.5 (see end of doc.) even if other public options are ticked]
    - Public building
    - Public road
    - Bridge
    - Park or countryside
    - Other: …………….

**1.4 Has anyone ever intervened in some way when you were suicidal [in a public place]** (for example by asking if you are ok, calling for help, or physically stopping you from attempting suicide?)

| Never  [Go to 1.4e] | Once | Between 2 and 4 times | Five times or more | n/a (nobody present) |
| --- | --- | --- | --- | --- |

**1.4b Who intervened? [Please tick as many as apply]:**

- A family member or partner/spouse

- A close friend
- An acquaintance, neighbour, school or work colleague
- A doctor or other health professional
- Emergency services
- A stranger
- Other. Please specify:

**1.4c Thinking back to a time [in a public place] that feels particularly significant to you, can you tell us a bit more about what happened?** *(For example: Where were you? How old were you at the time? Who intervened? How? Was anyone else present? What happened after? What was helpful? What was unhelpful?)*

*Please tell us about a specific event (If there is more than one you would be happy to share with us, there will be an option to do so later).*

**1.4d Are there any other times you would like to tell us about?** If so, please use the space below:

**1.4e Do you have any other experiences of being interrupted or distracted - by someone or something - when you were suicidal, in such a way that stopped or delayed you from attempting suicide at that point?**

**1.4f** **Has anything or anyone ever intervened or interrupted you when you were suicidal in such a way that it made things worse?**

[GO TO SECTION 2]

PART 2

***This section is about your experience of being around other people who might be suicidal.***

**2.1 Have you ever been in a situation when someone around you appeared to be suicidal?** *For example, a friend or loved one may have told you they were very low or suicidal, or you may have noticed some unusual or concerning behaviour in someone you knew or a stranger.*

Yes [continue below]/ No [GO TO 2.17]

**2.2 Have you ever experienced this in a public place (such as a railway station or a bridge)?**

No

Yes - > (please tick as many as apply)

- - - Train/tube station or tracks
    - Public building
    - Public road
    - Bridge
    - Park or countryside
    - Other: …………….

**2.3 What signs do you notice or look out for to try and establish if someone might be at risk of suicide in a public place?**

**2.4 How confident are you in your ability to identify situations where someone may be at risk of suicide in a public place?**

| Extremely confident | Somewhat confident | Neither confident nor not confident | Not very confident | Not at all confident |
| --- | --- | --- | --- | --- |

**2.5 How confident are you in your ability to intervene in situations where someone may be at risk of suicide in a public place?**

| Extremely confident | Somewhat confident | Neither confident nor not confident | Not very confident | Not at all confident |
| --- | --- | --- | --- | --- |

**2.6 Have you ever intervened in some way when someone around you appeared to be suicidal [in a public place / at a railway location], for example by talking to them, asking if they are ok, calling for help or attempting to interrupt or make contact with them in some other way?**

| No  [Go to 2.13] | Once | Between 2 and 4 times | Five times or more |
| --- | --- | --- | --- |

**2.7 Was the person at risk of suicide [please tick as many as apply]:**

- A family member or partner/spouse
- A close friend
- An acquaintance, neighbour, school or work colleague
- A client/patient
- A stranger
- Other. Please specify:

**2.8 Did your intervention involve [please tick as many as apply]:**

- Approaching the person and asking them directly about suicidal thoughts
- Approaching the person, but not asking directly about suicidal thoughts (e.g. making small talk)
- A non-verbal gesture (e.g. moving closer to the person, smiling or making eye contact)
- Calling for help
- Removing or preventing access to means of suicide (including by restraining the person)
- Other: please describe:

**2.9 Thinking back to a time when you intervened [in a public place/at a railway location] that feels particularly significant to you, can you please tell us a bit more about what happened?** *(For example: Where were you? Who was in danger? What did you do? Why? Was anyone else present? What happened after? What went well? Is there anything you wish you had done differently?)*

*Please tell us about a specific event (If there is more than one you would be happy to share with us, there will be an option to do so later).*

**2.10 Are there any other times you would like to tell us about, at similar or different locations?** If so, please use the space below:

**2.11 [ALL] Is there anything that would discourage or prevent you from intervening when someone appears to be suicidal in a public place?**

**2.12 Would any of these factors ever stopped you from intervening with someone at risk of suicide in a public place [if no at 2.2]**

**OR: Have any of these factors ever stopped you from intervening when someone appeared to be suicidal in a public place/at a railway location? [Please tick all that apply]**

- Worrying that it would make things worse
- Thinking the person would not want me to intervene
- Not knowing what to do or say
- Not knowing the person involved
- Thinking someone else would intervene
- Thinking I may have misjudged the situation
- Feeling embarrassed/uncomfortable
- Being concerned for my own safety or that of people around me
- Thinking it is not my responsibility
- Nobody else intervening
- Somebody else intervening, or about to do so
- Not being able to call for help
- Being too busy to intervene
- Things happening too quickly to intervene
- Thinking it is not serious enough to intervene
- Other. Please specify:

[GO TO PART 3]

**2.13** **What do you think discouraged or prevented you from intervening when someone around you was at risk of suicide [in a public place/at a railway location]?**

2.14 **Thinking back to a specific time [in a public place/at a railway location], please tell us a bit more about what happened.** (*For example: Where were you? Who was in danger? Was anyone else present? What stopped you from intervening? What happened after? What went well? Is there anything you wish you had done differently?)*

**2.15 Are there any other times you would like to tell us about, at similar or different locations?** If so, please use the space below:

**2.16 Have any of these factors influenced you to not intervene when someone appeared to be suicidal [in a public place/at a railway location]?** [Please tick all that apply]

- Worrying that it would make things worse
- Thinking the person would not want me to intervene
- Not knowing what to do or say
- Not knowing the person involved
- Thinking someone else would intervene
- Thinking I may have misjudged the situation
- Feeling embarrassed/uncomfortable
- Being concerned for my own safety or that of people around me
- Thinking it is not my responsibility
- Nobody else intervening
- Somebody else intervening, or about to do so
- Noticing someone else intervening
- Not being able to call for help
- Being too busy to intervene
- Things happening too quickly to intervene
- Thinking it is not serious enough to intervene
- Other. Please specify:

[GO TO PART 3]

**2.17 What sign or signs would you look out for to try and establish if someone might be at risk of suicide in a public place?**

**2.18 How confident are you in your ability to identify situations where someone may be at risk of suicide in a public place?**

| Extremely confident | Somewhat confident | Neither confident nor not confident | Not very confident | Not at all confident |
| --- | --- | --- | --- | --- |

2.19 **How confident are you in your ability to intervene in situations where someone may be at risk of suicide in a public place?**

| Extremely confident | Somewhat confident | Neither confident nor not confident | Not very confident | Not at all confident |
| --- | --- | --- | --- | --- |

**2.20 If you noticed that someone appeared upset or was in distress on a platform at a train station, what is the likelihood that you would intervene in some way *(for example by approaching the person, asking if they are ok, calling a member of staff, or attempting to interrupt or make contact with them in some other way)?***

| Extremely likely  1 | 2 | 3 | 4 | Extremely unlikely  5 |
| --- | --- | --- | --- | --- |

[if 1-3 to 2.20]:

**2.20a How do you think you might intervene, and why?**

**2.20b How would you NOT intervene, and why?**

[If 4 or 5 to 2.20]:

**2.20c What do you think would discourage or prevent you from intervening in a situation such as this?**

**2.20d Which of these factors, if any, would contribute to your decision not to intervene? [please tick as many as apply]**

- Worrying that it would make things worse
- Thinking the person would not want me to intervene
- Not knowing what to do or say
- Not knowing the person involved
- Thinking someone else would intervene
- Thinking I may have misjudged the situation
- Feeling embarrassed/uncomfortable
- Being concerned for my own safety or that of people around me
- Thinking it is not my responsibility
- Nobody else intervening
- Somebody else intervening, or about to do so
- Not being able to call for help
- Being too busy to intervene
- Things happening too quickly to intervene
- Thinking it is not serious enough to intervene
- Other. Please specify:

**2.21 What might make you more likely to intervene in a situation such as this?**

PART 3 [ALL]

**A1. In general, do you think it is possible to prevent suicide?**

| Always | Often | Sometimes | Rarely | Never |
| --- | --- | --- | --- | --- |

**A.1b Please explain why**

**A2. Do you believe it is safe to ask someone if they are feeling suicidal?**

| Always | Often | Sometimes | Rarely | Never |
| --- | --- | --- | --- | --- |

**A.2b Please explain why**

**A3 What do you think could be done to encourage more people to intervene in a safe and helpful way when someone is suicidal in a public place?**

**A4. What do you think are the most helpful things a person can say or do when someone around them is suicidal?**

**A5. What do you think are the least helpful things a person can say or do when someone around them is suicidal?**

***Finally, to help us understand how people’s responses vary across age, gender and other characteristics, we would be grateful if you could answer a few last questions:***

**3.1 How old are you?**

**3.2 How do you describe your gender?**

- Female
- Male
- Transgender
- Gender non-binary/non-conforming
- I describe my gender in another way (please state) [free text box]
- Prefer not to say

**3.3 Do you live in the UK?**

- No (Please specify if you wish.......)
- Yes -> - England (other than London)
  - - Ireland
    - London
    - Scotland
    - Wales

### **3.4 How do you describe your ethnicity?** [Please tick against one of the following]:

- Asian or Asian British (Please specify if you wish.......)
- Black or Black British (Please specify if you wish.......)
- Chinese or Other ethnic group (Please specify if you wish.......)
- Mixed (Please specify if you wish.......)
- White (Please specify if you wish.......)
- Prefer not to say

**3.5 What, if any, is your religion or belief?**

- No religion
- Buddhist
- Christian (including Church of England, Catholic, Protestant and all other Christian denominations)
- Hindu
- Jewish
- Muslim
- Sikh
- Other (Please specify if you wish):
- Prefer not to say

**3.6 What is your current occupation? ________________**

**3.7 Does your current or previous occupation involve working with people who are suicidal?** Yes/no

**3.8 Have you ever worked as a mental health professional?** Yes/no

Please specify if you wish:

**3.9 Do you have any connection with the rail industry (for example as a train operator, station employee or transport police officer?)** Yes/No

**3.10 Have you ever received any formal training in helping people who are suicidal?** Yes/no

Please specify if you wish:

**3.11 Are you familiar with Samaritans’ *Small Talk Saves Lives Campaign*?** [include link] Yes/no

**ADDITIONAL COMMENTS**

**Please use this space for any additional comments**

**SECTION 1.5. ADDITIONAL QUESTIONS RE: SUICIDALITY ON THE RAILWAYS**

***As one of the key aims of this research is to inform specific initiatives to prevent suicide in a railway context, it would be very helpful if you could answer some additional questions about your thoughts and experiences in relation to this.***

**To help us understand the context of your experiences, please indicate which of these best applies to you:**

- I have had thoughts of suicide on the railways, but never acted on these
- I have felt suicidal whilst on the railways (e.g. at a station or on a train), but my suicidal thoughts involved other methods and/or locations
- I have attempted suicide on the railways [->options below]
  - - Once*
    - between 2 and 4 times**
    - 5 times or more **

RAILWAY SUICIDAL THOUGHTS BUT NOT ACTED ON THEM

**1. Approximately how long ago did you have thoughts of suicide on the railways? _________________________**

**2. Why did you consider this particular method?**

**3. Are you familiar with anyone who has attempted or died by suicide on the railways?** Yes/no/unsure

[If yes -> please provide more information if you wish: ……]

**4. At the time of considering suicide on the railways, were you aware of other suicides or attempted suicides involving this method…** [please tick all that apply]:

- By hearing or reading about it in the news
- By reading about it online
- By hearing about an incident (or incidents) at a station or on a train (e.g., when a delay was announced because of a person being hit by a train)
- Through your local community and/or social circles
- Other:
- **Did any of these factors influence your thoughts about attempting suicide on the railways?**

**5. At the time, how frequently did you travel by train or tube?**

- Daily
- At least weekly
- At least once a month
- Less than once a month

**6. Did you research this method online?**

Yes/no/unsure

**7. Did your thoughts of suicide on the railways involve a specific location or context? If so, what do you think influenced this?**

**8. Have you had thoughts of suicide on the London Underground?**

**Yes/No/Unsure**

**If yes -> what influenced you to attempt suicide at this location rather than on a main railway line?**

**9. Do you have any connection with the rail industry (e.g. through your work), or any particular associations you make with the railways (e.g. specific memories or images)?**

10**. What did you think were the chances you would die if you attempted to take your life on the railways?**

[5 point slider: Extremely likely – extremely unlikely; I don’t know]

**11. What did you think were the chances you would be stopped or interrupted in some way, if you attempted to take your life on the railways?**

[5 point slider: Extremely likely – extremely unlikely]

- **Did this influence what you did or didn’t do in any way?**
- **Who did you think might intervene?**
- **Did anyone intervene or interrupt you in any way when you had thoughts of suicide on the railways?** Yes/no
- If so, please provide more information *(For example: Where were you? How old were you at the time? Who intervened? How? Was anyone else present? What happened after? What was helpful? What was unhelpful?)*

*Please tell us about a specific event (If there is more than one you would be happy to share with us, there will be an option to do so later).*

**Are there other times you would like to tell us about?** If so, please use the space below:

**12. Do you have any other experiences of being interrupted or distracted - by someone or something - when you were suicidal, in such a way that stopped or delayed you from attempting suicide at that point?**

**13. Has anything or anyone ever intervened or interrupted you when you were suicidal in such a way that it made things worse?**

**14. What prevented you from attempting suicide at a railway location?**

**15. Have you ever attempted suicide by other methods?** Yes/no

- Once
- between 2 and 4 times
- more than 5 times

Please provide more information if you wish:

**16. How does attempting suicide on the railways compare to other methods you have used or thought about using?**

**17. What do you think the rail industry can do to prevent suicide attempts on the railways?**

**18. What could make things worse?**

**19. Is there anything else you would like to add in relation to this?**

[GO TO SECTION 2]

SUICIDAL ON THE RAILWAYS, BUT NO THOUGHTS OF RAIL SUICIDE

**1. Did your suicidal thoughts involve a specific method or location?** Please provide more information if you wish

1. **Did you at any point consider but then discard the possibility of attempting suicide on the railways? Yes/no/unsure**

**If yes: Why did you decide against this method?**

[Then go to 1.4 (if no other public location ticked) or 1.5]

FOR THOSE WHO MADE SUICIDE ATTEMPTS ON THE RAILWAYS

***Approximately how long ago did you attempt suicide on the railways?**

****When did you last attempt suicide on the railways?**

**2. Why did you use this particular method?**

**3. At the time, what did you think were the chances you would die as a result of your attempt?**

[5 point slider: Extremely likely – extremely unlikely; I don’t know]

**4. To what extent had you planned your attempt?**

[5 point slider: Not at all - I had planned it in detail]

**5. Was anybody present or nearby at the time of your attempt?**

Yes/no/unsure

Please specify if you wish:

**6. What did you think were the chances you would be stopped or interrupted in some way, when you attempted to take your life on the railways?**

[5 point slider: Extremely likely – extremely unlikely]

- **Did this influence what you did or didn’t do in any way?**
- **Who did you think might intervene?**
- **Did you take any steps to avoid being stopped or interrupted?**

Yes/no/unsure - Please provide more information if you wish

- **Did anyone intervene or interrupt you in any way when you attempted suicide on the railways?** Yes/no
- If so, please provide more information *(For example: Where were you? How old were you at the time? Who intervened? How? Was anyone else present? What happened after? What was helpful? What was unhelpful?)*

*Please tell us about a specific event (If there is more than one you would be happy to share with us, there will be an option to do so later).*

**Are there other times you would like to tell us about?** If so, please use the space below:

**Do you have any other experiences of being interrupted or distracted - by someone or something - when you were suicidal, in such a way that stopped or delayed you from attempting suicide at that point?**

**Has anything or anyone ever intervened or interrupted you when you were suicidal in such a way that it made things worse?**

**7. Are you familiar with anyone who has attempted or died by suicide on the railways?** Yes/no/unsure [please provide more information if you wish: ……]

**8. At the time of your attempt or attempts on the railways, were you aware of other suicides or attempted suicides involving this method…** [please tick all that apply]:

- By hearing or reading about it in the news
- By reading about it online
- By hearing about an incident (or incidents) at a station or on a train (e.g., when a delay was announced because of a person being hit by a train)
- Through your local community and/or social circles
- Other:
- **Did any of these factors influence your decision to attempt suicide on the railways?**

**9. Did you research this method online before your attempt(s)?**

Yes/no/unsure

**10. Is there anything in particular that triggered your attempt or attempts on the railways?**

**11. Were you under the influence of alcohol or drugs at the time?**

Yes/no/unsure

**12. Looking back, do you think there was anything in your behaviour or demeanor that a passerby could have noticed, suggesting that you were planning to take your own life?**

**13. What might a passerby or member of staff have done to interrupt or prevent you from attempting suicide on that occasion?**

**14. Did anything else, or could anything else have interrupted or prevented your attempt?**

**15. What happened after your attempt?**

**16. What do you think influenced the specific railway location of your attempt (or attempts)?**

**17. Where were you when you attempted suicide (e.g. station platform, railway bridge, etc.)?**

**18. How close was this to where you lived at the time?**

- Within a mile
- Between 1 and 5 miles
- Between 5 and 10 miles
- Between 10 to 50 miles
- Over 50 miles away

**19. At the time of your attempt/s, how frequently did you travel by train or tube?**

- Daily
- At least weekly
- At least once a month
- Less than once a month

**20. Do you have any connection with the rail industry (e.g. through your work), or any particular associations you make with the railways (e.g. specific memories or images)?**

**20b. Have any of you ever attempted suicide on the London Underground [->what influenced you to attempt suicide at this location rather than on a main railway line?]**

**21. Have you ever attempted suicide by other methods?** Yes/no

- Once
- between 2 and 4 times
- more than 5 times

Please provide more information if you wish:

**22. How does attempting suicide on the railways compare to other methods you have used or thought about using?**

**23. What might have discouraged you from attempting suicide at a railway location?**

**24. What can the rail industry do to prevent suicide attempts on the railways?**

**25. What could make things worse?**

**26. Is there anything else you would like to add in relation to this?**
